# Autophagy‐mediated activation of the AIM2 inflammasome enhances M1 polarization of microglia and exacerbates retinal neovascularization

**DOI:** 10.1002/mco2.668

**Published:** 2024-07-29

**Authors:** Xianyang Liu, Qian Zhou, Jiayu Meng, Hangjia Zuo, Ruonan Li, Rui Zhang, Huiping Lu, Zhi Zhang, Hongshun Li, Shuhao Zeng, Meng Tian, Hong Wang, Ke Hu, Na Li, Liming Mao, Shengping Hou

**Affiliations:** ^1^ The First Affiliated Hospital of Chongqing Medical University Chongqing China; ^2^ Chongqing Key Laboratory of Ophthalmology Chongqing China; ^3^ Sichuan Provincial Key Laboratory for Human Disease Gene Study Sichuan Provincial People's Hospital University of Electronic Science and Technology of China Chengdu China; ^4^ Department of Ophthalmology Qilu Hospital Cheeloo College of Medicine Shandong University Jinan China; ^5^ Beijing Institute of Ophthalmology Beijing Tongren Eye Center Beijing Ophthalmology & Visual Sciences Key Laboratory Beijing Tongren Hospital Capital Medical University Beijing China; ^6^ Department of Laboratory Medicine, Beijing Tongren Hospital Capital Medical University Beijing China; ^7^ Department of Immunology School of Medicine Nantong University Nantong China

**Keywords:** AIM2 inflammasomes, autophagy, microglia, retinal angiogenesis, retinopathy of prematurity

## Abstract

Retinopathy of prematurity (ROP) is a retinal neovascularization (RNV) disease that is characterized by abnormal blood vessel development in the retina. Importantly, the etiology of ROP remains understudied. We re‐analyzed previously published single‐cell data and discovered a strong correlation between microglia and RNV diseases, particularly ROP. Subsequently, we found that reactive oxygen species reduced autophagy‐dependent protein degradation of absent in melanoma 2 (AIM2) in hypoxic BV2 cells, leading to increased AIM2 protein accumulation. Furthermore, we engineered AIM2 knockout mice and observed that the RNV was significantly reduced compared to wild‐type mice. In vitro vascular function assays also demonstrated diminished angiogenic capabilities following AIM2 knockdown in hypoxic BV2 cells. Mechanistically, AIM2 enhanced the M1‐type polarization of microglia via the ASC/CASP1/IL‐1β pathway, resulting in RNV. Notably, the administration of recombinant protein IL‐1β exacerbated angiogenesis, while its inhibition ameliorated the condition. Taken together, our study provides a novel therapeutic target for ROP and offers insight into the interaction between pyroptosis and autophagy.

## Graphical abstract

Under hypoxia, autophagy is impaired, leading to increased AIM2 protein accumulation. AIM2 promotes M1‐type activation of BV2 cells and enhances the expression of ASC, CASP1, and IL‐1β, consequently aggravating angiogenesis.

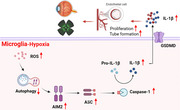

## 1 INTRODUCTION

Retinopathy of prematurity (ROP) is a disorder characterized by retinal neovascularization (RNV) that has emerged as a prominent contributor to childhood blindness globally.^1^, ^2^ ROP typically presents as retinal ischemia, pathological angiogenesis, and fibrous tissue hyperplasia, leading subsequently to retinal detachment and eventually irreversible blindness.^3^ Anti‐vascular endothelial growth factor (VEGF) drugs have been widely used in clinical practice to inhibit the growth of neovascularization in ROP. However, their efficacy is limited to a partial blockade of angiogenesis.^4^, ^5^ Furthermore, it has been reported that repeated intravitreal injections of VEGF in infants should be cautiously considered in light of the potential systemic effects, including impacts on the cardiovascular system and the developing nervous system, due to the important physiological role VEGF plays during infant development.^6^, ^7^, ^8^ Presently, there remains an urgent need for a safe and effective therapy for ROP.

Researchers have recently investigated the role of immune‐mediated inflammation in the initiation, progression, and treatment of RNV diseases.^9^ Microglia, as resident immune cells in the retina, play a crucial role in both the immune response and angiogenesis.^10^, ^11^ It has been reported that microglia interact with endothelial cells to form neurovascular units (NVUs) during the early stages of central nervous system development, which supports blood vessel formation and integrity.^12^, ^13^ Gao et al. found that retinal vascularization is reduced following selective clearance of microglia by PLX3397 or clodronate liposomes.^14^ These studies highlight the pivotal role of microglia in angiogenesis.

Microglia can detect and respond to danger signals and pathogen‐associated molecular patterns (PAMPs) through pattern recognition receptors (PRRs), such as Toll‐like receptors, NOD‐like receptors (NLRs), and absence in melanoma 2 (AIM2).^15^, ^16^ Upon activation, microglia can become polarized and produce a variety of pro‐inflammatory cytokines and pro‐angiogenesis factors that contribute to neuroinflammation and the promotion of vessel formation.^17^, ^18^ Furthermore, the activation of the inflammasome in microglia can amplify the inflammatory response, leading to the release of mature IL‐1β and IL‐18.^19^, ^20^

In ROP, oxidative stress occurs at an early stage, leading to the activation of molecular patterns associated with cellular damage as well as extensive DNA strand breaks and damage. AIM2, an intracellular DNA sensor, represents a deficiency in one of the PRRs, thereby exerting a substantial influence on the modulation of the innate immune response.^21^, ^22^ The C‐terminal region of AIM2 can recognize double‐stranded DNA (dsDNA) and other ligands. Additionally, AIM2 recruits the downstream molecule apoptosis‐associated speck‐like protein containing a CARD (ASC). In this process, ASC acts as an important bridging molecule that recruits the effector protein CASP1 into the inflammasome complex.^23^ The activation of AIM2 brings CASP1 molecules in close proximity to each other, thus producing autocatalytically active CASP1 molecules. Then, the precursor cell factors IL‐1β and IL‐18 are cleaved and subsequently secreted extracellularly.^24^ Previous studies reported that intercellular adhesion molecule 1 was regulated by IL‐1β, resulting in neovascularization.^25^ However, the involvement of AIM2 in angiogenesis has yet to be explored.

In this study, we developed an oxygen‐induced retinopathy (OIR) mouse model that is commonly used to study ROP. To investigate the role of microglia‐mediated pyroptosis in OIR, we examined the expression of inflammasome‐related genes, including *Nlrp1*, *Nlrp3*, *Nlrc4*, *Nlrp6*, *Nlrp12*, and *Aim2*. Notably, we discovered that while *Aim2* mRNA expression was significantly decreased in OIR, its protein level was significantly increased. Interestingly, it was found that under hypoxic conditions, autophagy was inhibited, resulting in an increase in AIM2 protein level. Furthermore, we engineered AIM2 knockout (AIM2 KO) mice and found that the neovascularization in AIM2 KO mice with OIR was attenuated. A series of genetic and functional deletion experiments in BV2 cells revealed that silencing AIM2 inhibited microglial M1‐type activation and reduced the expression of ASC, CASP1, IL‐18, and IL‐1β, which ultimately attenuated angiogenesis. Collectively, our data suggest that the AIM2/ASC/CASP1/IL‐1β microglial pyroptosis axis represents a potential anti‐angiogenic therapy for ROP.

## 2 RESULTS

### 2.1 Single‐cell analysis reveals a strong association between microglia and RNV

**FIGURE 1:**
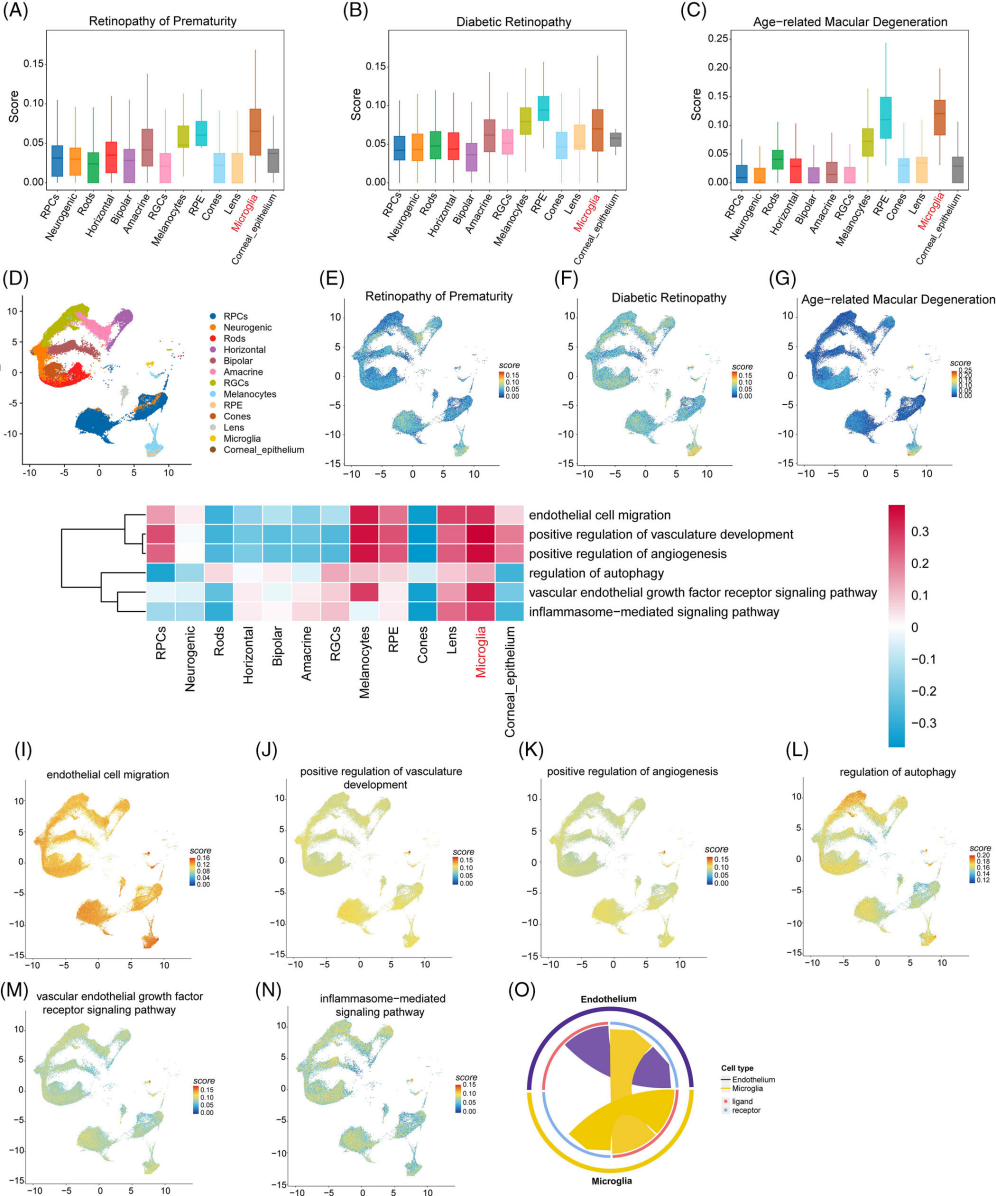
Single‐cell analysis shows the correlation of microglia and retinopathy of prematurity (ROP) disease. (A‒C) UCell images showing the relative expression of concerned eye disease‐related genes in different cell types. (D‒G) Uniform manifold approximation and projection (UMAP) and FeaturePlot of ROP, diabetic retinopathy (DR), and age‐related macular degeneration (AMD). (H‒N) Gene set variation analysis (GSVA) analysis and FeaturePlot of microglia. (O) Microglia and endothelial cells communication.

To clarify the pathology of RNV diseases, we analyzed the single‐cell data from our previous study (GSE228370).^26^ It was found that microglia were strongly associated with RNV diseases, including ROP, diabetic retinopathy (DR), and age‐related macular degeneration (AMD); this effect was especially pronounced for ROP (Figure 1A–C). In addition, analysis using uniform manifold approximation and projection (UMAP) revealed a significantly elevated score for microglia, indicating their prominent role in RNV diseases (Figure 1D–G). Gene set variation analysis (GSVA) analysis and the FeaturePlot showed that microglia were enriched in positive regulation of angiogenesis, inflammasome, and autophagy pathways (Figure 1H–N). Furthermore, the cell‒cell communication analysis showed that microglia interact closely with endothelial cells (Figures 1O and Figure S1A,B). Taken together, these results identify a pivotal role for microglia in angiogenesis.

### 2.2 AIM2 protein levels increased in the retinas of OIR mice and in hypoxic BV2 cells

**FIGURE 2:**
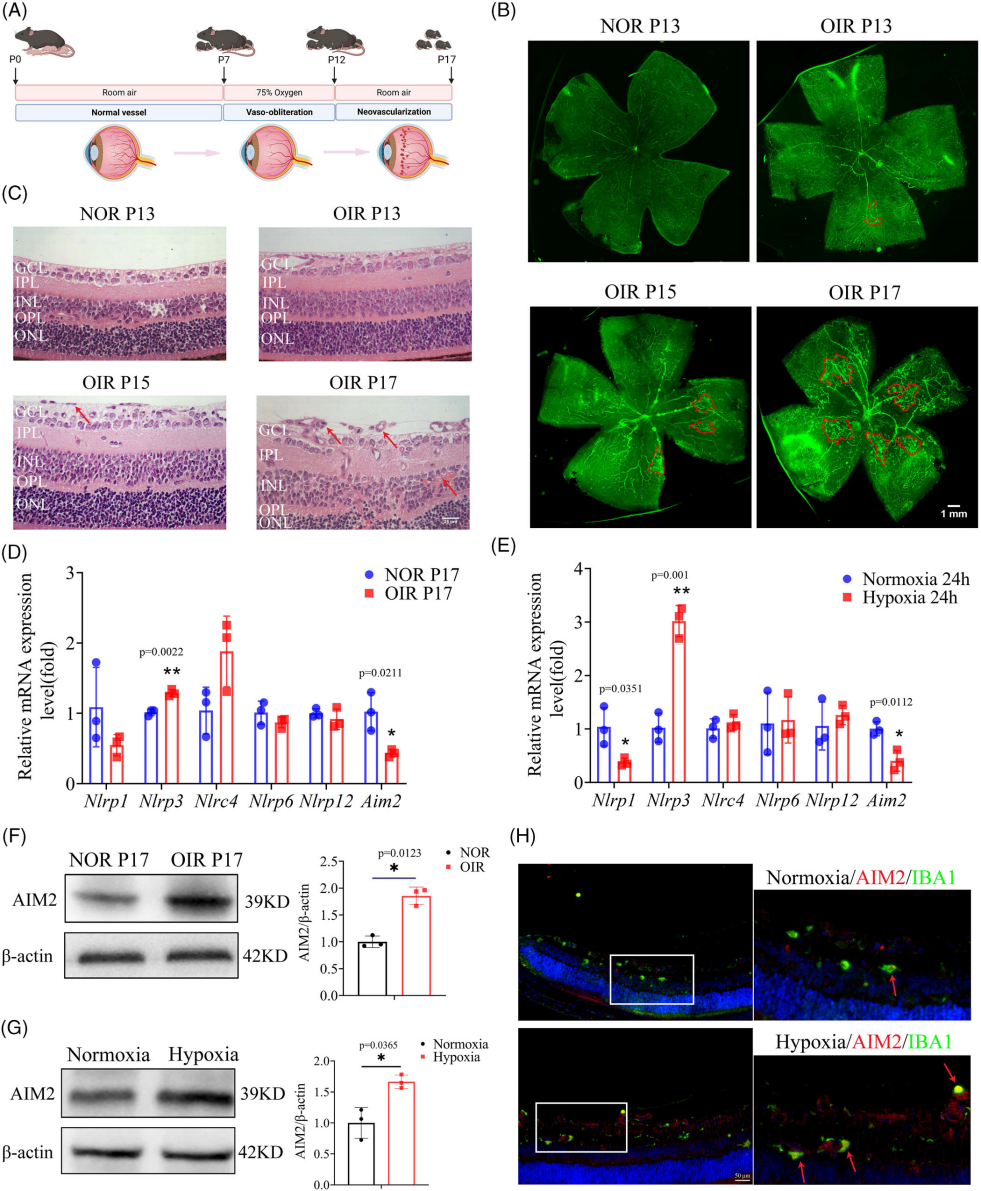
Absent in melanoma 2 (AIM2) expression is upregulated in the retinas of oxygen‐induced retinopathy (OIR) mice and in hypoxic BV2 cells. (A) The flowchart and pathological character of OIR mice model with time course. (B) Immunofluorescence images of retinal flat‐mounts in NOR and OIR mice stained CD31 (endothelial cell maker) at postnatal days 13, 15, and 17, neovascularization was circled in red. Scale bar: 1 mm. (C) Images of hematoxylin and eosin (H&E) staining in NOR and OIR mice at postnatal days 13, 15, and 17. Scale bar: 25 µm. (D) mRNA level of inflammasome including *Nlrp1*, *Nlrp3*, *Nlrp6*, *Nlrp12*, and *Aim2* between NOR and OIR retinas (mean ± standard deviation [SD]; *n* = 3/group; ^*^ *p* < 0.05, ^**^ *p* < 0.01, unpaired Student's *t*‐test). (E) mRNA expression of *Nlrp1*, *Nlrp3*, *Nlrp6*, *Nlrp12*, and *Aim2* in BV2 cells treated with hypoxia compared to that with normoxia (mean ± SD; *n* = 3/group; ^*^ *p* < 0.05, ^**^ *p* < 0.01, unpaired Student's *t*‐test). (F) Protein expression and quantification of AIM2 between NOR and OIR retinas (mean ± SD; *n* = 3/group; ^*^ *p* < 0.05, unpaired Student's *t*‐test). (G) Protein level and quantitative graph of AIM2 in BV2 cells treated with hypoxia compared to those treated with normoxia (mean ± SD; *n* = 3/group; ^*^ *p* < 0.05, unpaired Student's *t*‐test). (H) Representative immunofluorescence images of retinal slices in NOR and OIR mice stained with IBA1 and AIM2. Scale bar: 50 µm. NOR, normoxia.

We designed a flowchart schematizing the OIR experiment and the phenotypic characteristics of angiogenesis (Figure 2A). Retinal flat‐mount images were taken using a confocal microscope on postnatal days 13 (P13), 15 (P15), and 17 (P17). Prolonged hypoxia resulted in aggravated neovascularization, which peaked on P17 (Figure 2B), consistent with previous results.^27^, ^28^ Supporting these findings, hematoxylin and eosin (H&E) staining revealed that prolonged hypoxia resulted in an increased number of cells breaking through the internal limiting membrane, reaching the maximum on P17 (Figure 2C). To further investigate the involvement of inflammasomes during peak angiogenesis, we used RT‐qPCR to assess the mRNA expression levels of inflammasome‐related genes, including *Nlrp1*, *Nlrp3*, *Nlrc4*, *Nlrp6*, *Nlrp12*, and *Aim2*, in OIR P17 retinas. We found that the expression of *Nlrp3* was significantly increased, while that of *Aim2* was significantly decreased (Figure 2D). Likewise, we examined the mRNA expression of the same genes in both hypoxia‐treated and normoxia‐treated microglia (BV2 cells) and found an increase in *Nlrp3* and a decrease in *Nlrp1* and *Aim2* in the hypoxic cells (Figure 2E).

Because the role of NLRP3 in retinal angiogenesis has been well described, we focused on exploring the effect of AIM2 in OIR, which remains understudied and unclear. Western blot analysis revealed that AIM2 protein levels were significantly upregulated in OIR retinas and in hypoxic BV2 cells with hypoxia (Figure 2F,G). Furthermore, immunofluorescence of retinal sections showed higher expression levels of AIM2 in microglia from OIR mice than from normoxia (NOR) mice (Figure 2H). Additionally, subcellular localization experiment showed that AIM2 was expressed in both the nucleus and cytosol, and there were no significant changes in the subcellular localization between normoxic and hypoxic BV2 cells (Figure S2). Our results suggest that the inflammasome AIM2 may be instrumental in the development of OIR.

### 2.3 Hypoxia‐induced autophagy dysfunction leads to increased AIM2 protein levels in BV2 cells

**FIGURE 3:**
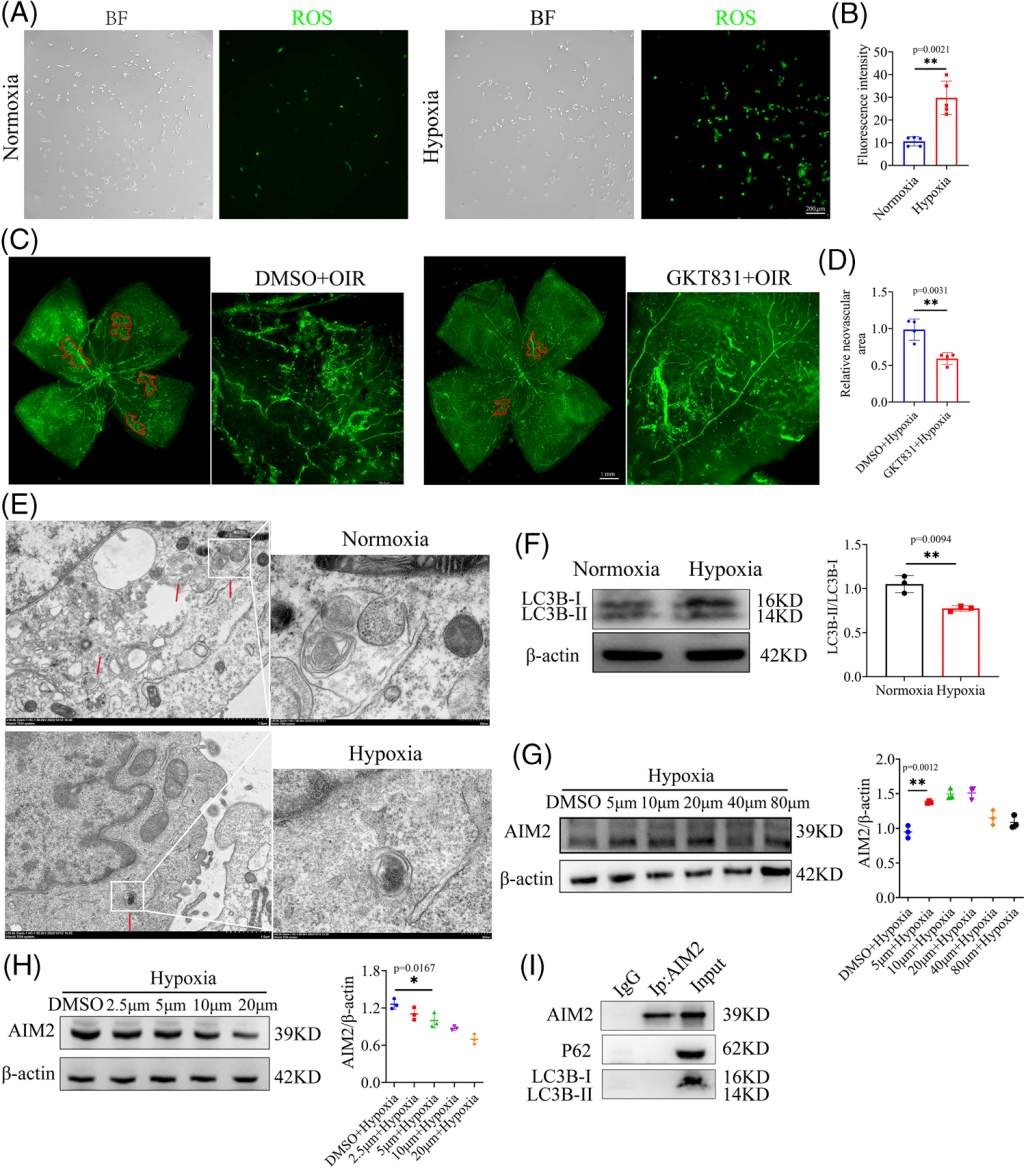
Autophagy regulates the protein level of absent in melanoma 2 (AIM2). (A and B) Reactive oxygen species (ROS) levels of BV2 cells under normoxia or hypoxia (mean ± standard deviation [SD]; *n* = 5/group; ^**^ *p* < 0.01, unpaired Student's *t*‐test; scale bar: 200 µm). (C and D) Retinal neovascularization (RNV) area in oxygen‐induced retinopathy (OIR) with DMSO or GKT831 (mean ± SD; *n* = 4/group; ^**^ *p* < 0.01, unpaired Student's *t*‐test; scale bar: 1 mm). (E) Images of transmission electron microscopy (TEM) between normoxic and hypoxic BV2 cells. Scale bar: 1 µm, 200 nm. (F) Protein expression and quantification of autophagy marker LC3B in normoxic and hypoxic BV2 cells (mean ± SD; *n* = 3/group; ^*^ *p* < 0.05, ^**^ *p* < 0.01, unpaired Student's *t*‐test). (G) Expression and quantification of AIM2 after disposing chloroquine at concentrations of 5, 10, 20, 40, and 80 µm (mean ± SD; *n* = 3/group; ^**^ *p* < 0.001, one‐way analysis of variance [ANOVA]). (H) Protein expression and quantification of AIM2 in hypoxic BV2 cells treated with rapamycin (mean ± SD; *n* = 3/group; ^*^ *p* < 0.05, one‐way ANOVA). (I) Co‐immunoprecipitation (Co‐IP) of AIM2 with LC3B or P62. DMSO, Dimethyl Sulfoxide.

Intriguingly, we noted a substantial reduction in the mRNA expression of AIM2, as illustrated in Figure 2D,E, whereas its protein level was significantly increased, as shown in Figure 2F–H. We speculate that this was due to reduced AIM2 protein degradation under hypoxic conditions, ultimately resulting in an accumulation of AIM2 protein. Autophagy is a highly sensitive cellular process that mediates protein degradation, and can be regulated by oxidative stress.^29^, ^30^, ^31^ In hypoxic BV2 cells, a markedly elevated level of reactive oxygen species (ROS) was observed (Figure 3A,B). Setanaxib (GKT831) is a potent and highly selective NOX1 and NOX4 inhibitor that significantly reduces the production of ROS.^32^, ^33^ Intravitreal injection with Setanaxib resulted in a marked reduction in neovascularization compared to the Dimethyl Sulfoxide (DMSO) group in the OIR model (Figure 3C,D).

Additionally, through transmission electron microscopy (TEM), we found that autophagosomes were destroyed in hypoxic BV2 cells (Figure 3E). Western blot analysis was used to detect the protein level of LC3B, a widely used autophagy marker, in hypoxic and normoxic BV2 cells; in agreement with our previous results, LC3B was significantly decreased in the hypoxic group (Figure 3F). To confirm the influence of autophagy on AIM2 expression, we stimulated hypoxic BV2 cells with the autophagy inhibitor chloroquine at 5, 10, 20, 40, and 80 µm. Cell viability assay demonstrated that approximately 90% of the cells were viable (Figure S3A). Western blot analysis revealed that AIM2 protein levels increased in a concentration‐independent manner following the inhibition of autophagy (Figure 3G). Rapamycin, recognized as an autophagy activator, effectively and specifically suppresses the mTOR signaling pathway.^34^, ^35^ We applied rapamycin in an attempt to restore autophagy activation in hypoxic BV2 cells and found that the protein level of AIM2 was downregulated (Figure 3H). To investigate any reciprocal influence of AIM2, we examined the expression of ATG5, Beclin1, and LC3B in hypoxic BV2 cells with AIM2 knockdown (KD); however, the results indicated that AIM2 does not regulate the autophagy process (Figure S3B).

To evaluate whether the autophagy marker LC3B or P62 directly interacts with AIM2, we conducted co‐immunoprecipitation (Co‐IP) experiments. The results indicated that there is no direct binding relationship between these proteins (Figure 3I). This finding implies that autophagy may exert its regulatory effects on AIM2 expression through indirect pathways rather than through direct protein‒protein interactions.

### 2.4 Inhibition of AIM2 attenuates RNV

**FIGURE 4:**
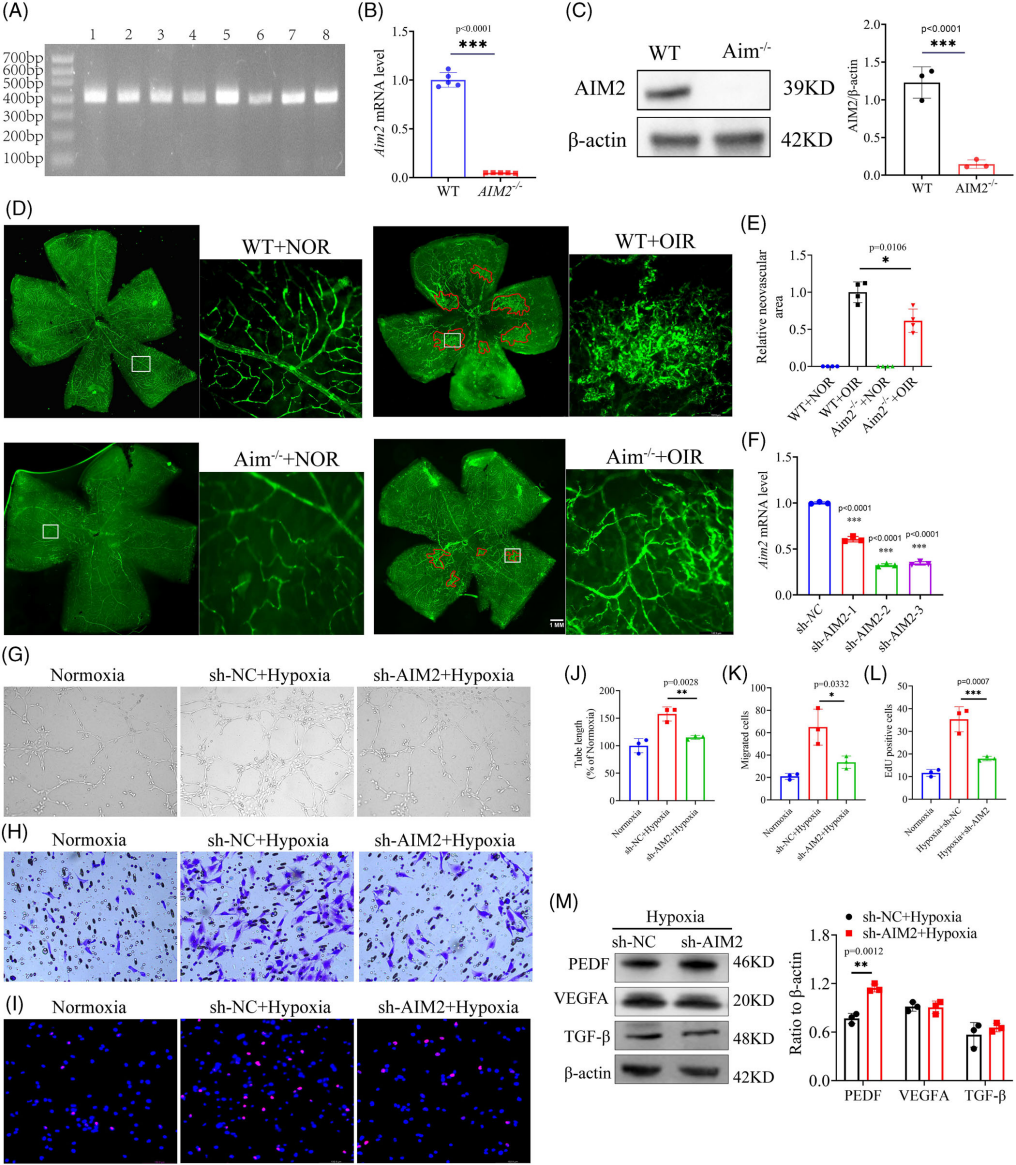
Silencing of absent in melanoma 2 (AIM2) alleviates neovascularization in vivo and in vitro. (A) PCR and gel electrophoresis of homozygous AIM2 knockout mice (*n* = 8). (B) mRNA expression of AIM2 knockout efficiency (mean ± standard deviation [SD]; *n* = 3/group; ^***^ *p* < 0.001, unpaired Student's *t*‐test). (C) Protein level of AIM2 knockout efficiency (mean ± SD; *n* = 3/group; ^***^ *p* < 0.001, unpaired Student's *t*‐test). (D) Immunofluorescence images of retinal flat‐mounts, the neovascularization was circled in red. Scale bar: 1 mm. (E) Quantitative graph of angiogenic area (mean ± SD; *n* = 4/group; ^*^ *p* < 0.05, one‐way analysis of variance [ANOVA]). (F) mRNA expression of AIM2 in BV2 cells after transfection with negative control (NC), shAIM2‐1, shAIM2‐2, or shAIM2‐3 lentivirus (mean ± SD; *n* = 3/group; ^***^ *p* < 0.001, one‐way ANOVA). (G and J) Representative images and quantification of in vitro tube formation analysis (mean ± SD; *n* = 3/group; ^*^ *p* < 0.05, one‐way ANOVA). (H and K) Transwell migration assay and quantitative graph of murine endothelial cells (MECs) after co‐culture with AIM2 knockdown BV2 cells (mean ± SD; *n* = 3/group; ^*^ *p* < 0.05, one‐way ANOVA). (I and L) 5‐Ethynyl‐2ʹ‐deoxyuridine (EdU) assay was used to analyze the proliferation of MECs after co‐culture with AIM2 knockdown BV2 cells (mean ± SD; *n* = 3/group; ^**^ *p* < 0.01, one‐way ANOVA). (M) The protein level of pigment epithelium‐derived factor (PEDF), vascular endothelial growth factor A (VEGFA), and transforming growth factor‐β (TGF‐β) in AIM2‐silenced hypoxic BV2 cells (mean ± SD; *n* = 3/group; ^**^ *p* < 0.01, unpaired Student's *t*‐test).

To further investigate the role of AIM2 in RNV, we constructed AIM2 KO mice. PCR and gel electrophoresis confirmed the presence of the homozygous AIM2 KO mice at 400 bp (Figure 4A). RT‐qPCR showed that the knockout efficiency reached ∼90% (Figure 4B). Additionally, Western blot assay showed almost no bands in the electrophoresis the lane of AIM2 KO group (Figure 4C). Retinal flat‐mount images showed that the extent and leakage of RNV were alleviated in AIM2 KO mice with OIR than in wild type (WT) mice with OIR (Figure 4D,E).

Subsequently, a series of functional assays were performed in vitro. We transfected BV2 cells with AIM2 KD lentivirus, and the resulting images showed a transfection efficiency of more than 80% (Figure S4A). RT‐qPCR analysis revealed that the mRNA knockdown efficiency of lentiviral siAIM2‐2 and siAIM2‐3 constructs was about 70% (Figure 4F), while Western blot analysis showed that the protein knockdown efficiency of lentiviral siAIM2‐3 was about 70% (Figure S4B). BV2 cells were transfected with the lentiviral siAIM2‐3 for subsequent experiments and were screened by puromycin dihydrochloride (2 µg/mL) to obtain a stable transgenic strain.

A co‐culture system was designed in an effort to investigate the effects of AIM2 in BV2 cells on murine endothelial cells (MECs). In this system, monolayer MECs were seeded in an apical chamber and BV2 cells were seeded in a basolateral chamber (Figure S4C). MECs co‐cultured with hypoxic BV2 cells had enhanced tube‐forming capacity, whereas MECs with AIM2 KD had significantly weakened tube‐forming capacity (Figure 4G,J). Additionally, MECs co‐cultured with hypoxic BV2 cells possessed significantly improved migration and proliferation capabilities, which was diminished following knockdown of the AIM2 gene (Figure 4H,I,K,L). We conducted Western blot to examine the protein levels of angiogenic factors VEGFA, PEDF, and TGF‐β in AIM2‐silenced hypoxic BV2 cells. Our findings indicated that AIM2 had no effect on the expression of VEGFA and TGF‐β, but it significantly increased the PEDF protein level (Figure 4M). Collectively, our findings suggest that AIM2 promotes RNV in OIR.

### 2.5 Inhibition of microglial AIM2 expression attenuates cell proliferation and polarization

**FIGURE 5:**
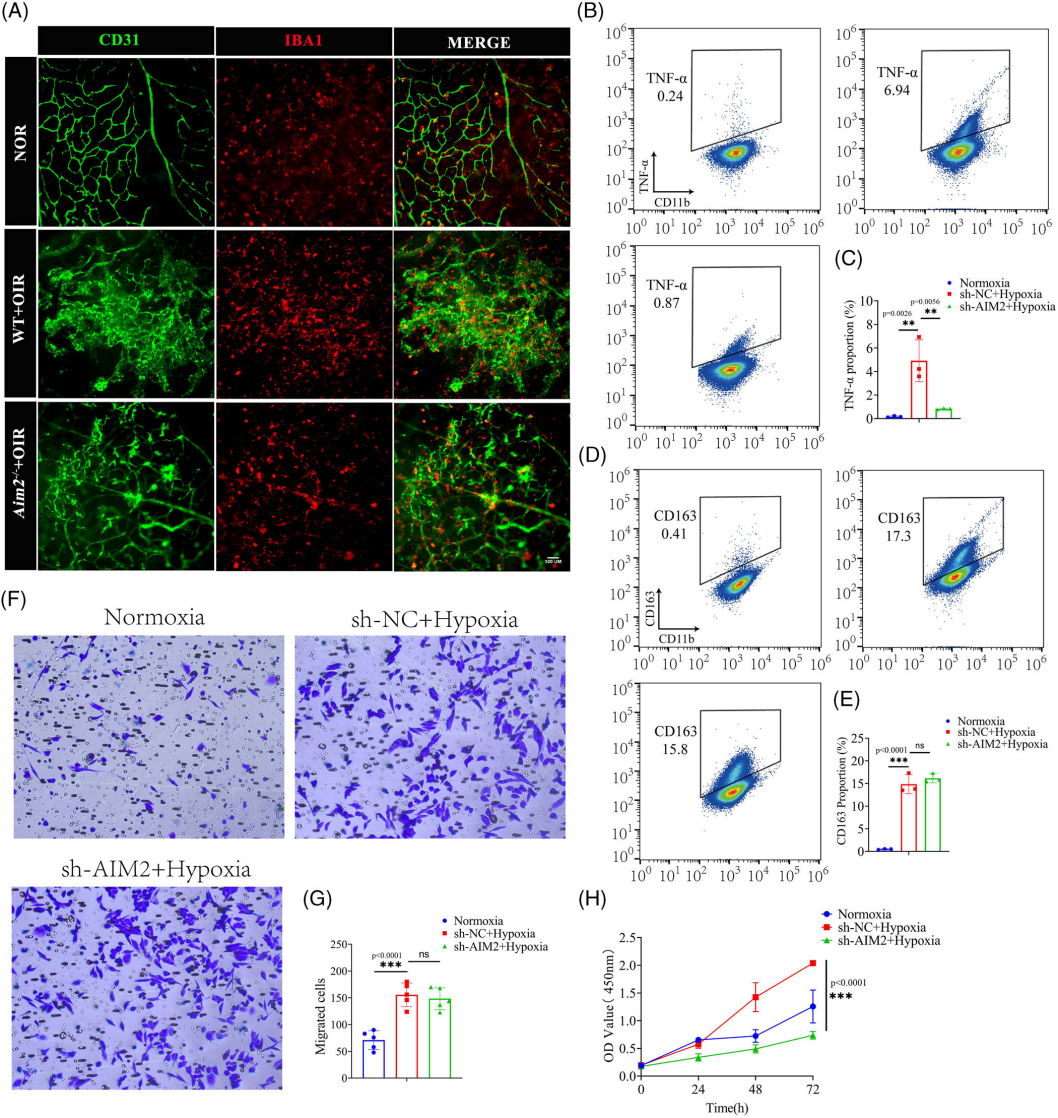
Absent in melanoma 2 (AIM2) inhibition alters the abilities of microglial migration and polarization, not proliferation. (A) Immunofluorescence images of retinal flat‐mounts stained with CD31 and IBA1. Scale bar: 100 µm. (B‒E) Flow cytometry analysis of M1‐type maker (TNF‐α) and M2‐type maker (CD163) in normoxic BV2 cells, sh‐NC hypoxic BV2 cells, and sh‐AIM2 hypoxia BV2 cells (mean ± standard deviation [SD]; *n* = 3/group; ns > 0.05, ^**^ *p* < 0.01, ^***^ *p* < 0.001, one‐way analysis of variance [ANOVA]). (F and G) Transwell assay and quantitative graph of BV2 cells under hypoxia with sh‐NC or sh‐AIM2 or normoxia (mean ± SD; *n* = 3/group; ns > 0.05, ^***^ *p* < 0.001, one‐way ANOVA). (H) Proliferation of BV2 cells under hypoxia with sh‐NC or sh‐AIM2 or normoxia (mean ± SD; *n* = 6/group; ^***^ *p* < 0.001, one‐way ANOVA).

Microglia are highly plastic and heterogeneous cells that play a crucial role in immune surveillance and microenvironmental stabilization through changes in polarization, proliferation, migration, and migration.^10^, ^36^, ^37^ However, the influence of inflammasome AIM2 on microglia still remains unclear. In images of NOR retinas, the cell bodies of microglia were small and branched; in contrast, in OIR retinas, microglial cell bodies were enlarged, with shorter protrusions, and amoeboid. However, there were no significant microglial morphological changes between WT and AIM2 KO mice with OIR (Figure 5A).

We subsequently examined the expression of TNF‐α (M1‐type markers) and CD163 (M2‐type markers) in BV2 cells using flow cytometry. This revealed that under hypoxic conditions, the expression levels of TNF‐α and CD163 were all significantly increased, suggesting that BV2 cells were activated and underwent polarization. Following AIM2 inhibition and subsequent hypoxia, we observed a significant reduction in the expression of TNF‐α; however, no significant changes were detected in the expression levels of CD163 (Figure 5B–E).

Transwell assay revealed that the migration ability of BV2 cells was enhanced under hypoxia, whereas there was no change in migration ability when AIM2 was knocked down (Figure 5F,G). Cell Counting Kit‐8 (CCK‐8) assay showed a significant increase in the number of BV2 cells under hypoxic conditions compared to normoxic conditions, and a decrease in the number of BV2 cells after AIM2 silencing (Figure 5H). Collectively, our findings suggest that inhibition of AIM2 attenuates microglial M1‐type activation and proliferation.

### 2.6 AIM2 regulates the protein levels of ASC, CASP1, and IL‐1β

**FIGURE 6:**
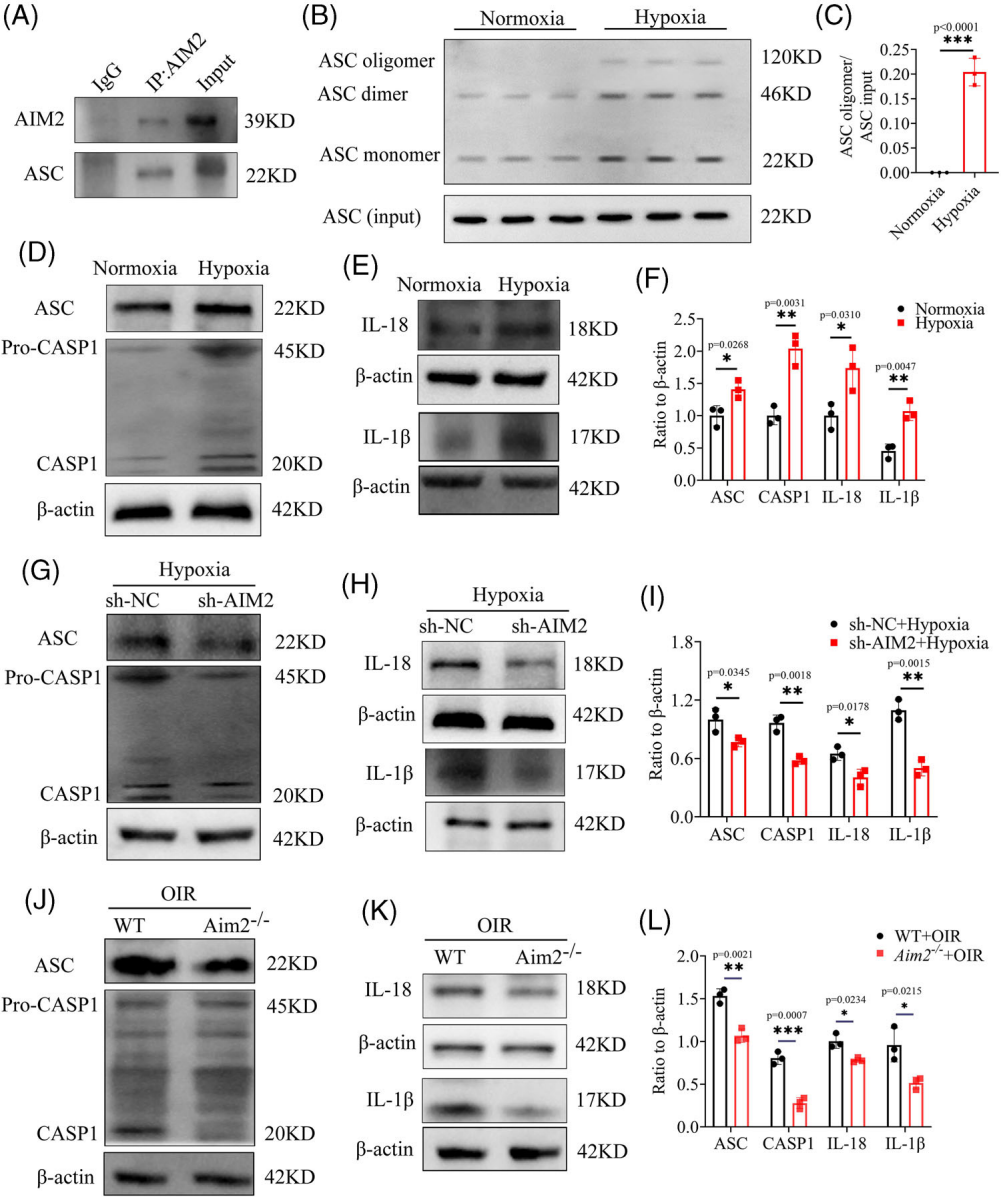
Absent in melanoma 2 (AIM2) regulates the ASC/CASP1/IL‐1β pathway. (A) Co‐immunoprecipitation (Co‐IP) of AIM2 with ASC. (B and C) Protein level and quantification ASC oligomer (mean ± standard deviation [SD]; *n* = 3/group; ^***^ *p* < 0.001, unpaired Student's *t*‐test). (D‒F) Protein level and quantification of ASC, CASP1, IL‐18, and IL‐1β in BV2 cells treated with hypoxia compared to those treated with normoxia (mean ± SD; *n* = 3/group; ^*^ *p* < 0.05, ^**^ *p* < 0.01, unpaired Student's *t*‐test). (G‒I) Protein level and quantitative graph of ASC, CASP1, IL‐18, and IL‐1β between sh‐NC and sh‐AIM2 BV2 cells under hypoxia (mean ± SD; *n* = 3/group; ^*^ *p* < 0.05, ^**^ *p* < 0.01, unpaired Student's *t*‐test). (J‒L) Expression of ASC, CASP1, IL‐18, and IL‐1β in Aim2 KO mice compared to wild type (WT) with OIR (mean ± SD; *n* = 3/group; ^*^ *p* < 0.05, ^**^ *p* < 0.01, ^***^ *p* < 0.01, unpaired Student's *t*‐test).

Previous research has demonstrated that both AIM2 and ASC have the pyrin structural domain, allowing them to combine and then initiate caspase 1 activation.^38^ To corroborate these findings, we performed the Co‐IP assay and demonstrated that AIM2 and ASC directly interacted with each other (Figure 6A). Additionally, we performed ASC oligomerization assay to show AIM2 inflammasome formation (Figure 6B,C) and then detected several classical downstream mediators of the inflammasome; that is, we found increased levels of ASC, cleaved CASP1, matured IL‐1β, and matured IL‐18 in hypoxia‐treated BV2 cells (Figure 6D–F). After inhibiting AIM2 in hypoxic BV2 cells under hypoxia, the expression of both ASC and CASP1 was significantly reduced, and the protein level of mature IL‐1β and mature IL‐18 also showed a similar trend between sh‐NC and sh‐AIM2 BV2 cells under hypoxia, which was especially pronounced in the case of mature IL‐1β (Figure 6G‒I).

Subsequently, we performed Western blotting on samples from retinas of WT and Aim2 KO mice with OIR. The results agreed with our previous findings, showing that the protein levels of ASC, cleaved CASP1, mature IL‐1β, and mature IL‐18 were decreased in retinas of Aim2 KO mice with OIR (Figure 6J–L).

### 2.7 Recombinant protein IL‐1β promotes retinal angiogenesis and inhibition of IL‐1β alleviates neovascularization

**FIGURE 7:**
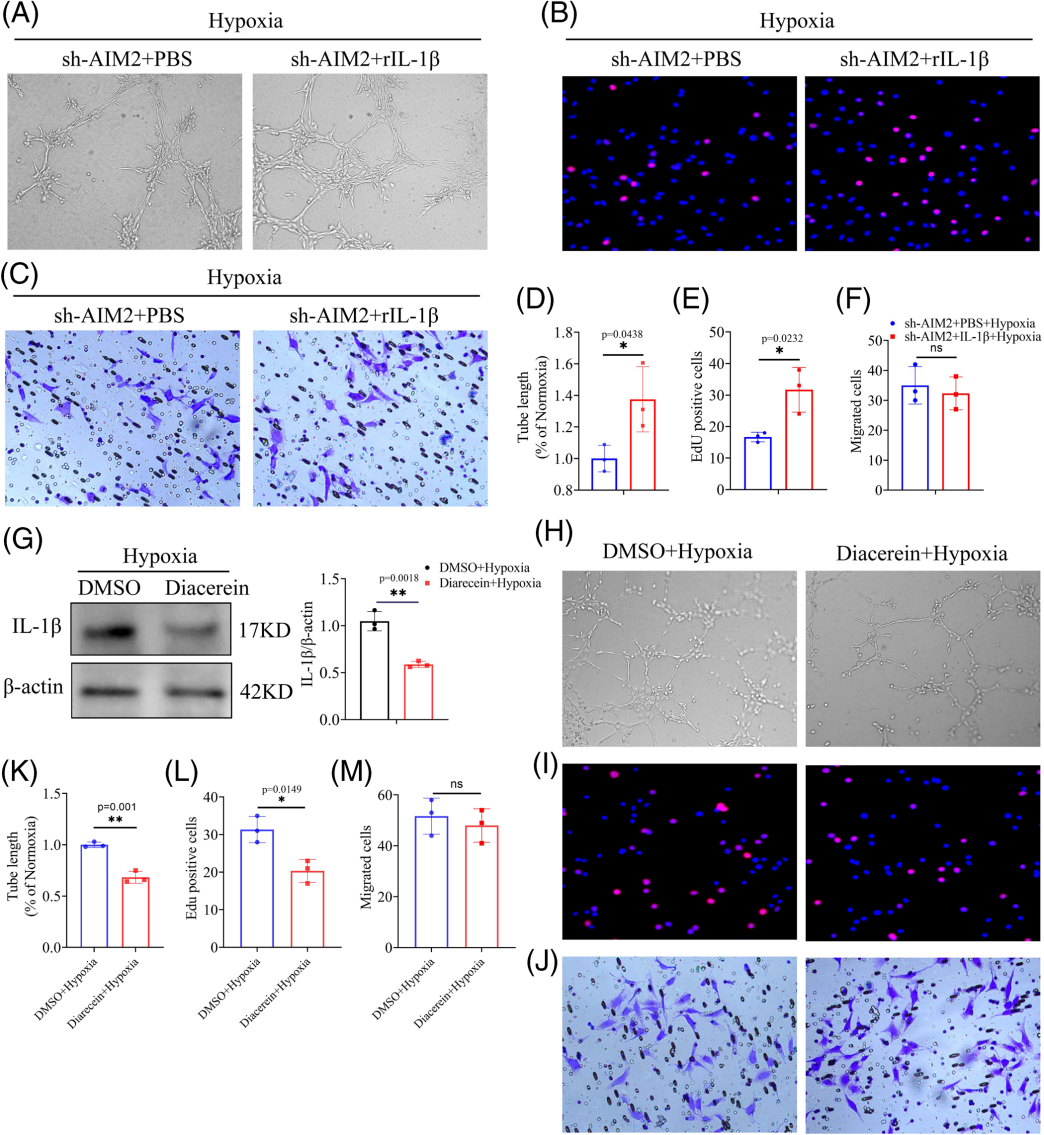
Supplement of IL‐1β promotes neovascularization and inhibition of IL‐1β alleviates it. (A and D) Tube formation of murine endothelial cells (MECs) co‐cultured with sh‐absent in melanoma 2 (AIM2) cells with PBS or rIL‐1β under hypoxia (mean ± standard deviation [SD]; *n* = 3/group; ^*^ *p* < 0.05, unpaired Student's *t*‐test). (B and E) 5‐Ethynyl‐2ʹ‐deoxyuridine (EdU) staining in the two groups mentioned above (mean ± SD; *n* = 3/group; ^*^ *p* < 0.05, unpaired Student's *t*‐test). (C and F) Migration ability in the above groups (mean ± SD; *n* = 3/group; ns > 0.05, unpaired Student's *t*‐test). (G) Protein level and quantification of IL‐1β in BV2 cells after treated with Diacerein (mean ± SD; *n* = 3/group; ^*^ *p* < 0.05, unpaired Student's *t*‐test). (H‒M) Abilities of tube formation, proliferation, and migration of MECs co‐cultured with hypoxic BV2 cells with or without Diacerein (mean ± SD; *n* = 3/group; ns > 0.05, ^*^ *p* < 0.05, unpaired Student's *t*‐test).

Previous studies have showed that IL‐1β modulates the expression of VEGFA by activating promoter binding of STAT3 and NF‐κB, leading to neovascularization.^39^ To confirm the role of effector IL‐1β in promoting retinal angiogenesis, the recombinant protein IL‐1β was applied to AIM2‐silenced hypoxic BV2 cells, which were then co‐cultured with MECs. We observed an enhancement in MECs tube formation by when co‐cultured with IL‐1β‐supplemented BV2 cells than when co‐cultured with PBS (Figure 7A,D). Similarly, the proliferation of MECs also increased following co‐culture with IL‐1β‐supplemented BV2 cells (Figure 7B,E). However, there was no significant change in MECs migration ability (Figure 7C,F).

Diacerein has been reported as a significant inhibitor of IL‐1β production.^40^ We then administrated BV2 cells with Diacerein, Western blot showed that the inhibited degree of mature IL‐1β reached around 50% (Figure 7G). In addition, we investigated whether Diacerein had an impact on AIM2 expression and discovered that it did not affect the protein level of AIM2 (Figure S5). Subsequently, a series of functional tests suggested that the inhibition of IL‐1β in hypoxic BV2 cells weakened the MECs tube‐forming and proliferation abilities, but it had no effect on the migration ability (Figure 7H–M). Collectively, our results suggested that AIM2 inflammasome drives microglial polarization and exacerbates RNV via the ASC/CASP1/IL‐1β signaling pathway.

## 3 DISCUSSION

RNV is a common ocular condition that threatens human vision and is a component of many diseases including ROP, AMD, and DR.^41^ Among these, ROP is one of the leading causes of childhood blindness worldwide.^42^, ^43^ Currently, methods to treat ROP produce only modest benefits; anti‐VEGFA and laser ablation therapy can temporarily control neovascularization, but the long‐term efficacy is limited, and repeated anti‐VEGF injection may cause VEGFA resistance and other side effects in children.^44^, ^45^ Therefore, more efficient and safer therapeutic strategies are urgently needed.

Hypoxia, which can aggravate retinal oxidative stress and promote retinal angiogenesis, is considered the main cause of ROP.^46^ Microglial cells, the resident immune cells in the retina, they can quickly respond to hypoxic conditions to maintain microenvironmental homeostasis, and they interact with endothelial cells to promote neovascularization.^47^, ^48^, ^49^ Accordingly, when PLX5622 or clodronic acid is used to selectively deplete microglia, neovascularization is effectively curbed.^50^ In our study, analysis of our previously published single‐cell data confirmed that microglial cells are strongly associated with ROP disease. In the OIR model, we found that microglial cells were highly proliferated and became activated in an amoeboid formation, converging around vascular endothelial cells. These results suggest that microglia possess highly plastic abilities and play a role in RNV through interactions with the vasculature.

Previous research has found that hypoxia initiates the RIP1/RIP3 signaling pathway, which mediates microglial necrosis and increases the release of FGF2, leading to a high degree of RNV; after inhibiting RIP1/RIP3, retinal angiogenesis was effectively reduced.^48^ Pyroptosis is one of the most important cell death types affecting microglia, which regulates both their plasticity and polarity.^51^ However, the relationship between microglial death and inflammasomes in ROP remained unclear. To address this knowledge gap, here, we examined the expression of inflammasome‐forming genes, including *Nlrp1*, *Nlrp3*, *Nlrp6*, *Nlrp12*, and *Aim2*, in hypoxia‐treated microglia. Interestingly, we observed that hypoxia was associated with a reduction in the mRNA expression of *Aim2*, while its protein level was increased. To delve deeper into the role of AIM2 in OIR, we generated Aim2 KO transgenic mice and induced an OIR model. Retinal flat‐mount image analysis revealed a significant attenuation of angiogenesis in Aim2 KO mice. In our in vitro experiments, MECs co‐cultured with AIM2‐inhibited BV2 cells also showed a reduction in tube formation, migration, and proliferation abilities.

Inflammasomes are multiprotein complexes that can sense and target PAMPs and molecular signals related to cell damage.^52^, ^53^ AIM2, as a DNA sensor, it can specifically recognize and detect impaired DNA molecules, such as DNA and damaged DNA in the cytoplasm resulting from the destruction of nuclear membrane integrity.^54^ N‐terminal pyrin domain (PYD) and C‐terminal domain comprise the main structure of AIM2. After binding to dsDNA, the AIM2 oligomer is formed, and the ligand ASC is aggregated under the interaction of PYD‒PYD; the aggregation of ASC leads to the activation of CASP1 and IL‐1β.^55^, ^56^, ^57^ ROS accumulation damages intracellular components and produces mass dsDNA, which is quickly recognized by and activates AIM2. Activated AIM2 can mediate the production of downstream inflammatory factors. In our experiments, the expression of ASC, cleaved CASP1, mature IL‐1β, and mature IL‐18 was increased under hypoxia; after silencing AIM2, they were significantly reduced. IL‐1β as a downstream effector can promote blood vessel formation.^58^, ^59^ Previous studies have reported that IL‐1β activates vascular adhesion molecules and promotes the secretion of VEGFA, leading to neovascularization.^39^

Notably, we observed that while hypoxia reduced AIM2 mRNA expression, yet increased its protein level both in vivo and in vitro. We hypothesize that this may be due to impaired AIM2 protein degradation under hypoxic conditions. Autophagy is one of the most important intracellular protein degradation methods,^60^ and it can be regulated by oxidative stress. Moderate oxidative stress rapidly activates autophagy to clear damaged proteins and repair tissues; yet excessive oxidative stress leads to impaired autophagy.^61^, ^62^ To validate the regulatory association between autophagy and AIM2, we treated hypoxic BV2 cells with the autophagy inhibitor chloroquine. The results demonstrated that the protein expression of AIM2 increased in a concentration‐independent manner. This innovative approach establishes a link between autophagy and pyroptosis, elucidating the regulatory influence of autophagy on AIM2. As an additional consideration, it is possible that feedback mechanisms are at play, which modulate the cellular response to hypoxic stress. These mechanisms could lead to an increase in AIM2 protein levels, serving as a compensatory response to the observed decrease in mRNA levels. Further studies will be conducted in the future to delve deeper into this phenomenon.

Moreover, rapamycin, an autophagy activator, was used to restore the autophagy activation in our experimental setup. Our results demonstrated that this intervention significantly reduced the protein levels of AIM2 in hypoxic BV2 cells. Previous studies discovered that rapamycin can bind with FKBP12 and specifically inhibit the mTOR signaling pathway, a crucial regulator of various cellular processes including autophagy, cell growth, and metabolism.^63^, ^64^ Given the established role of mTOR in autophagy regulation, we hypothesized that the mTOR pathway might be involved in modulating the activity of the inflammasome. To advance our understanding, we plan to conduct further studies that will provide molecular details of how the mTOR pathway affects the inflammasome. Additionally, we plan to intravitreally autophagy activator in the OIR model to assess the influence of activated autophagy on neovascularization. These future investigations will be instrumental in elucidating the complex signaling networks of autophagy and inflammation in OIR.

However, this study has several limitations. Our findings showed that the protein levels of both NLRP3 and AIM2 are elevated in the OIR. This suggests that both proteins may play a role in the progression of OIR disease, but the specific contributions of these proteins to the observed phenotypes remain unclear. To address this knowledge gap, we plan to generate NLRP3 KO mice, thereby allowing us to investigate the specific contributions of these proteins to the development of OIR and to determine which, if either, plays a more pivotal role in the disease process.

Additionally, given the specific contexts within and between species, across gender, developmental stage, complex tissue layers, and microglial states ultimately determine the phenotype and function of cells.^65^ In our study, we used the microglial cell line BV2 to investigate the underlying mechanisms; the BV2 cell line cannot reflect all the information of primary microglia. It may not fully represent the complexity and heterogeneity of microglia in the OIR disease state. Finally, the in vitro nature of BV2 cells may not fully recapitulate the in vivo microenvironment, which includes interactions with other cell types and the extracellular matrix. To address these limitations, future research could employ primary microglia isolated from retinas to validate our findings. In our study, we constructed AIM2 KO mice, which cannot completely demonstrate the role of AIM2 in microglia in OIR progression; in a future study, we will consider constructing conditional KO mice. Aside from this, we found that autophagy can regulate AIM2, but we did not identify which domain, terminal, or structure of AIM2 autophagy specifically recognizes; this is a goal of our next in‐depth research project.

In conclusion, our study discovered a novel link between autophagy and the AIM2 inflammasome, revealing that autophagy regulates AIM2 protein levels. We also established evidence that AIM2 plays a crucial role in promoting neovascularization through the ASC/CAPS1/IL‐1β pathway. These findings provide a potential therapeutic target and strategy for the management of ROP.

## 4 MATERIALS AND METHODS

### 4.1 Animals

The C57BL/6J mice employed in this investigation were obtained from the Experimental Animal Center at Chongqing Medical University. AIM2 KO mice were generously provided by Prof. Xiaopeng Qi. Approval for all experiments involving animals was obtained from the Ethics Committee of the First Affiliated Hospital of Chongqing Medical University (approval number: 2021‐612).

### 4.2 OIR model

To establish the OIR model in mice, P7 WT and Aim2 KO mouse pups with their nursing mother were exposed in hyperoxia (75% O_2_) until P12, then they were returned to normoxia (∼21% O_2_) for 5 days, which induced a relative state of hypoxia. On P17, peak of disease,^28^, ^66^ they were sacrificed. Subsequently, their retinas were carefully isolated for subsequent experimental procedures. Since there were no appreciable disparities between the sexes, data were gathered from the results for both males and females.

### 4.3 Identification of Aim2 KO mice

Agarose gel electrophoresis was utilized to determine the genotype of Aim2 KO mice. Following the manufacturer's protocol, we prepared a lysis buffer consisting of 1 M NaOH and 0.5 M EDTA, as well as a neutralization buffer containing 40 mM Tris and concentrated hydrochloric acid to adjust the pH to 4. Mice tails were collected and stored in labeled 1.5 mL centrifuge tubes. After incubation with 100 µL of lysis buffer at 95°C for 1 h, 100 µL of neutralization buffer was added to the centrifuge tubes, which were then centrifuged at 12,000 rpm for 3−5 min to obtain the supernatant for PCR. Each PCR reaction contained 10 µL of GoTaq Green Master Mix (M7122, Promega), 3 µL of primer mix consisting of 1 µL of each primer, and 7 µL of the supernatant from the mixed buffer. The PCR cycling conditions were as follows: initial denaturation at 94°C for 3 min, followed by 35 cycles of denaturation at 94°C for 30 s, annealing at 63°C for 30 s, and extension at 72°C for 40 s, with a final extension at 72°C for 10 min and a hold at 4°C indefinitely. The PCR products were subjected to agarose gel electrophoresis and visualized under UV fluorescence. The Aim2 KO mice displayed a single band of 400 bp.

### 4.4 Cell culture and reagents

Microglia cell line BV2 cell was purchased from ATCC. MEC was procured from FuHeng Biology. They were cultured in Dulbecco's Modified Eagle Medium F‐12 (DMEM‐F12) contained with 10% fetal bovine serum (FBS). Cells treated with 21% O_2_ defined as normoxia, and cells treated with 2% O_2_ for 24 h were defined as hypoxia in vitro. Autophagy inhibitor chloroquine was purchased from TargetMol (T8689); proteasome inhibitor MG132 was purchased from TargetMol (T2154); recombinant protein IL‐1β was purchased from SinoBiological (50101‐MNAE); and Diacerein was purchased from Selleck (S4267). Rapamycin was purchased from MedChemExpress (HY‐10219). Setanaxib was purchased from Selleck (S7171).

### 4.5 Quality control, dimension reduction, and clustering

Seurat v 3.1.2 was used for quality control, dimensionality reduction, and clustering. For each sample dataset, we filtered expression matrix by the following criteria: (1) cells with gene count less than 200 or with top 2% gene count were excluded; (2) cells with top 2% UMI count were excluded; (3) cells with mitochondrial content >20% were excluded; and (4) genes expressed in less than five cells were excluded. Gene expression matrix was normalized and scaled using functions NormalizeData and ScaleData. Top 2000 variable genes were selected by FindVariableFeatures for PCA analysis. Cells were separated into 13 clusters by FindClusters. Cell clusters were visualized using *t*‐distributed stochastic neighbor embedding or UMAP with Seurat functions RunTSNE and RunUMAP.

### 4.6 UCell gene set scoring

Gene set scoring was carried out using the R package UCell v 1.1.0.^67^ UCell scores are based on the Mann‒Whitney *U* statistic, which ranks query genes in order of their expression levels in individual cells. Since UCell is a rank‐based scoring method, it is suitable for use in large datasets containing multiple samples and batches.

### 4.7 Differentially expressed genes analysis

To identify differentially expressed genes (DEGs), we employed the Seurat FindMarkers function based on the Wilcoxon rank sum test with default parameters. Genes expressed in more than 10% of the cells in both compared groups and with an average log(fold change) value greater than 0.25 were selected as DEGs. The adjusted *p*‐value was calculated using Bonferroni correction, and a value of 0.05 was used as the criterion to assess statistical significance.

### 4.8 Pathway enrichment analysis

To investigate the potential functions, Gene Ontology (GO) analysis was used with the “clusterProfiler” R package v 3.16.1.^68^ Pathways with *p* adj value less than 0.05 were considered as significantly enriched. For GSVA pathway enrichment analysis, the average gene expression of each cell type was used as input data.^69^

### 4.9 Cell‐type annotation

The identification of the cell type for each cluster was determined based on the expression of canonical markers in the reference database SynEcoSysTM (Singleron Biotechnology). SynEcoSysTM contains a collection of canonical cell‐type markers for single‐cell sequencing data from CellMakerDB, PanglaoDB, and recently published literature.

### 4.10 Cell‒cell interaction analysis: CellPhoneDB

Cell‒cell interaction between microglia and endothelial cells was predicted based on known ligand‒receptor pairs using CellPhoneDB (v 2.1.0) version.^70^ The permutation number for calculating the null distribution of average ligand‒receptor pair expression in randomized cell identities was set to 1000. The individual ligand or receptor expression was thresholded by a cutoff based on the average log gene expression distribution for all genes across each cell type. Predicted interaction pairs with a *p*‐value <0.05 and an average log expression >0.1 were considered significant and visualized by dot plot in CellPhoneDB.

### 4.11 Cell Counting Kit‐8 assay

Briefly, in 96‐well microplates (Corning, Inc.), BV2 cells were seeded and cultivated in 100 µL medium with 5 × 10^3^ cells per well.^71^ Each well received 10 µL CCK‐8 reagent (MA0218, MeilunBio) under dark condition and cultured for 3 h. The optical density of individual wells was ascertained using a microplate reader obtained from ThermoFisher Scientific. The absorbance values served as indicators of cellular proliferation.

### 4.12 Transwell assay

Following a 24‐h hypoxia treatment, BV2 cells were seeded in the lower chamber of a 24‐well transwell plate (8 µm; Corning, Inc.) at a density of 5 × 10^4^ cells per well, using DMEM‐F12 supplemented with 10% FBS. Subsequently, MECs were seeded into the upper chambers at the same cell density with DMEM‐F12 containing 0.5% FBS. After co‐culturing for 24 h, the upper chambers were disassembled. MECs were immobilized using a 4% paraformaldehyde fix solution for 15 min, followed by three washes. The cells were then stained with 1% crystal violet. Utilizing a cotton swab, cells on the upper surface of the chamber membrane were wiped away, and images of the migrated cells on the lower surface of the chamber membrane were captured using a fluorescence microscope (Leica).

### 4.13 5‐Ethynyl‐2ʹ‐deoxyuridine staining

BV2 cells were subjected to 24 h of hypoxic conditions and then placed in the upper chambers (0.4 µm; Corning, Inc.) at 5 × 10^4^ cells per well, MECs were seeded into the basolateral chambers at 5 × 10^4^ cells per well. After 24 h of coculture, remove apical chambers, MECs were incubated with 20 µL of 5‐ethynyl‐2ʹ‐deoxyuridine (EdU) (Beyotime) for 1 h. Following this, cellular fixation was carried out using a 4% paraformaldehyde fixative solution for a duration of 10 min, succeeded by permeabilization with 3‰ Triton X‐100 for 30 min. Following this, cells were exposed to a 200 µL reaction mixture from the EdU kit for 30 min and subsequently stained with 1× Hoechst 33342 for 5 min. Imaging was conducted using a fluorescence microscope (Leica).

### 4.14 Tube formation assays

Tube formation experiment was performed according to the method of EdU assay. Matrigel basement membrane matrix (Corning) was applied to each well of a 96‐well plate for 50 µL. A total of 2 × 10^4^ suspended MECs were put on the well. Images were taken using microscope after an incubation period 6 h at 37°C, and then examined using ImageJ software.

### 4.15 Transmission electron microscopy

The morphology and number of autophagosome were observed through a transmission electron microscope. Briefly, normoxic and hypoxic BV2 cells were digested using 0.25% trypsin (Gibco) and washed with PBS for three times, then fixed with 2.5% glutaraldehyde. Following with exposing to 1% osmium acid for 1 h, cells were dehydrated using gradient ethanol (65%−90%), next, they were embedded with epoxy resin and were made into 50 nm slices. The ultrathin sections were stained with lead citrate and 1% uranyl acetate, then examined using a transmission electron microscope (HT7700). TEM resolution ranged between 1 µm and 200 nm.

### 4.16 Detection of intracellular ROS level

BV2 cells were counted with 2 × 10^5^ and planted in six‐well plates. The control group was cultured with normoxia for 24 h, while the experimental group was incubated at a hypoxic condition. Then, added 1 mL of diluted DCFH‐DA (10 mmol/L) to per well and incubated for 15 min. Next, the cells were added with medium to neutralize DCFH‐DA. Pictures were captured using a fluorescence microscope (Leica).

### 4.17 Hematoxylin and eosin staining

Eyeballs were fixed with FAS eyeball fixation solution (G1109, Servicebio) and embedded in paraffin according to standard methods. Briefly, retina was treated with graded alcohols (70%‒100%), washed with xylene and then embedded in paraffin. Microtomes were used to slice the paraffin blocks into 5‐µm sections, which were deparaffinized by xylene and rehydrated by different concentration of alcohols (70%‒100%). Subsequently, routine H&E staining was applied. The resulting sections were then observed and captured using a microscope.

### 4.18 Real‐time quantitative PCR

After the medium of cultured cell was removed, 1 mL of Trizol (Roche) was directly added to the lysed cells in a 3.5 cm diameter petri dish and followed with the manufacturer's instructions. Extracted RNA was reversed into cDNA using RT Master Mix (AG11705, Accurate Biotechnology [Hunan] Co., Ltd.), and the resulting cDNA was mixed with SYBR Green qPCR Master Mix, protected from light (AG11708, Accurate Biotechnology [Hunan] Co., Ltd.). mRNA expression levels were assessed using the ABI 7500 Real‐Time PCR System (Applied Biosystems). All primers were synthesized by Shanghai Sangon Co., Ltd., and their details are presented in Table S1. The mRNA expression was standardized with β‐actin and calculated using the 2^−ΔΔCT^ method.

### 4.19 Western blotting

BV2 cells were seeded in six‐well plate (Jet Biofl), after different treatment with them, following two washes with PBS, pre‐cooled buffer was added to lyse the retina or BV2 cells. The protein concentration in the retina or BV2 cells was determined using the Bicinchoninic Acid Kit (Beyotime). Subsequently, the protein was separated by gel electrophoresis and transferred onto a polyvinylidene fluoride (PVDF) membrane. The membrane underwent blocking with Fast Blocking Western reagent (Yeasen). Gently, added TBST to the wash the PVDF membrane, and the membrane were placed into a box at 4°C with primary antibodies for overnight incubation. Ultimately, visualization of the protein bands was achieved through the utilization of an ECL kit (KF8001, Affinity), and quantification was performed using ImageJ software. The primary antibodies used in this study are listed below: AIM2, 1:1000, Affinity; iNOS: 1:800, Abcam; TNF‐α, 1:1000, Abcam; CD206, 1:800, Proteintech; ARG1, 1:800, Proteintech; ASC, 1:1000, Abcam; CASP1, 1:650, Abcam; IL‐1β, 1:750, Abcam; IL‐18, 1:800, Abcam; LC‐3B, 1:600, Affinity; P62, 1:800, Abcam; β‐actin, 1:5000; Affinity. ATG5, 1:1000, Proteintech; Beclin1, 1:800, Affinity; VEGFA, 1:1000, Affinity; PEDF, 1:600, Huabio; TGF‐β, 1:1000, Bioss.

### 4.20 Co‐immunoprecipitation

BV2 cells were cultured according to the method described above. Co‐IP was performed using Thermo Scientific Pierce Co‐IP kit (ThermoFisher Scientific) according to the manual instruction. The lysate was added to the cell culture plate for full cell lysis at 4°C. The lysate was centrifuged at 12,000 rpm for 10 min, and the supernatant was collected and incubated with anti‐AIM2 antibody (63660, Cell Signaling Technology) or anti‐IgG antibody (30000‐0‐AP, Proteintech) at 4°C overnight. After binding with protein A/G Sepharose beads, the protein was eluted with SDS loading buffer and detected by Western blotting.

### 4.21 ASC oligomerization assay

BV2 cells were plated onto six‐well plates at a density of 2 × 10^6^ cells per well. They were then subjected to normoxic or hypoxic conditions for 24 h. After treatment, the supernatant was discarded, and 200 µL of pre‐cooled % NP40 (ThermoFisher) was added to each well. The cells were scraped off and transferred to a 1.5 mL centrifuge tube. The tubes were then placed on ice for 20 min and homogenized by passing through a 7‐gauge needle 10 times. The lysates were centrifuged at 4°C and 6000 rpm for 10 min. The supernatant was collected and the pellet was washed three times with 500 µL of pre‐cooled PBS. After washing, the pellet was resuspended in 500 µL of PBS. Freshly prepared 2 mM DSS crosslinker (ThermoFisher) was added and the mixture was incubated at 37°C for 30 min. Following incubation, the mixture was centrifuged again at 4°C and 6000 rpm for 10 min. The pellet was resuspended in 20 µL of SDS loading buffer, heated at 100°C for 10 min, and then subjected to Western blot analysis.

### 4.22 Flow cytometry

BV2 cells subjected to various treatments were restimulated using the Cell Activation Cocktail (containing Brefeldin A; BioLegend, 423303) for a duration of 4 h at 37°C. Post‐restimulation, the cells were stained for surface markers using the APC/Cyanine7 anti‐mouse CD11b antibody (BioLegend, 101225). They were subsequently fixed using a fixation buffer (BioLegend, 420801) and permeabilized (BioLegend, 421002). The cells were then stained using specific antibodies for 40 min at 4°C. The antibodies used included the PerCP/Cyanine5.5 anti‐mouse TNF‐α (BioLegend, 506322) and the PE/Cyanine7 anti‐mouse CD163 (BioLegend, 156707). The stained cells were subsequently analyzed using the ThermoFisher Scientific Attune NxT flow cytometer, with data processing performed using the FlowJo software (FlowJo Co.).

### 4.23 Lentivirus transfection

A total of 2 × 10^5^ BV2 cells were seeded in per well of six‐well plates and cultured overnight. After the cells were adherent, AIM2 KD lentivirus was added according to the appropriate multiplicity of infection in DMEM‐F12 with 10% FBS. After 8 h, according to the cell status, medium with virus was replaced with fresh complete medium. After transfection, the cell growth was observed, and medium containing puromycin (2 µg/mL) was added for selection to obtain stable cell lines.

### 4.24 Immunofluorescence staining and retinal flat‐mount

A total of 2 × 10^4^ cells were resuspended and counted, then seeded on the slides, and cultured in an incubator overnight. Immunostaining was performed according to the standard methods. Following fixation with 4% paraformaldehyde, BV2 cells were permeabilized using 0.5% Triton X‐100 (G1204, Servicebio) for 30 min. Subsequently, cells were blocked with goat serum at 37°C for 30 min, and the primary antibody was incubated overnight at 4°C. After that, cells were treated with corresponding secondary antibody for 1 h at room temperature. Lastly, the nuclear of cells were stained with 4,6‐diamidino‐2‐phenylindole. Images were captured by fluorescence microscope (Leica).

The eyes of mice were also fixed in 4% paraformaldehyde at room temperature for 2 h. After excision of surrounding tissues, the retina was dissected into a quadrifoliate configuration and mounted onto a glass slide. Following the permeabilization of cell membranes, the antigen was subsequently blocked with goat serum for 30 min at 37°C. Gently shake off the sealing solution, add PBS to the slices with a certain proportion of CD31 (1:350), and the slices are placed flat in a wet box at 4°C for overnight incubation. Pictures were taken under fluorescence microscope (Leica).

### 4.25 Statistics analysis

All experiments performed in this study were at least three independent replicates. The data were presented as mean ± standard deviation, and statistical analyses were performed utilizing SPSS Statistics 27 (IBM Corp.). Figures were generated using Prism 9.0 (GraphPad).

## AUTHOR CONTRIBUTIONS

Xianyang Liu, Qian Zhou, and Jiayu Meng designed the research and performed the study. Hangjia Zuo and Ke Hu revised the manuscript. Rui Zhang helped to polish the manuscript. Huiping Lu, Ruonan Li, and Hongshun Li helped to analyze the GEO data. Zhi Zhang, Meng Tian, Hong Wang, and Shuhao Zeng contributed reagents, materials, and analysis tools. Na Li, Liming Mao, and Shengping Hou helped to conceive the research and revise the manuscript. Authors have read and approved the final manuscript.

## CONFLICT OF INTEREST STATEMENT

All authors declare they have no competing financial interests and consent for publication.

## ETHICS STATEMENT

All experiments involving animals were approved by the Ethics Committee of the First Affiliated Hospital of Chongqing Medical University (approval number: 2021‐612).

## Supporting information

Supporting Information

## Data Availability

The authors declare that all the data supporting the findings of this study are available in the manuscript.
