# Supplementary material for: Autophagy‐mediated activation of the AIM2 inflammasome enhances M1 polarization of microglia and exacerbates retinal neovascularization
Source: MedComm (2020). 2024 Jul 29;5(8):e668. doi: 10.1002/mco2.668 (PMC11286542; doi:10.1002/mco2.668)
Supplement: Supplementary file 1 — Supporting Information [file MCO2-5-e668-s001.docx]

**Figure S1.** Cell communication of Microglia and Epithelium.

**Figure S2.** Subcellular localization of AIM2 between normoxic and hypoxic BV2 cells.

**Figure S3.** (A) Cell viability of BV2 cells treated with chloroquine. (B) The protein level and quantification of ATG5, Beclin1, and LC3B in AIM2 silenced hypoxic BV2 cells (mean ± SD; n = 3/group; *P < 0.05, unpaired Student’s t-test).

**Figure S4.** (A) Lentiviral transfection efficiency. (B) Protein level and quantification of AIM2 in BV2 cells transfected with NC, shAIM2-1, shAIM2-2 or shAIM2-3 lentivirus (mean ± SD; n = 3/group; ***P < 0.001, one-way ANOVA). (C) BV2 cells and MECs co-culture system.

**Figure S5.** The protein level of AIM2 in hypoxic BV2 cells with or without Diacerein (mean ± SD; n = 3/group; ns > 0.05, unpaired Student’s t-test.

**Table S1.** The Primers for RT-qPCR used in this study.

**Figure S1**

**
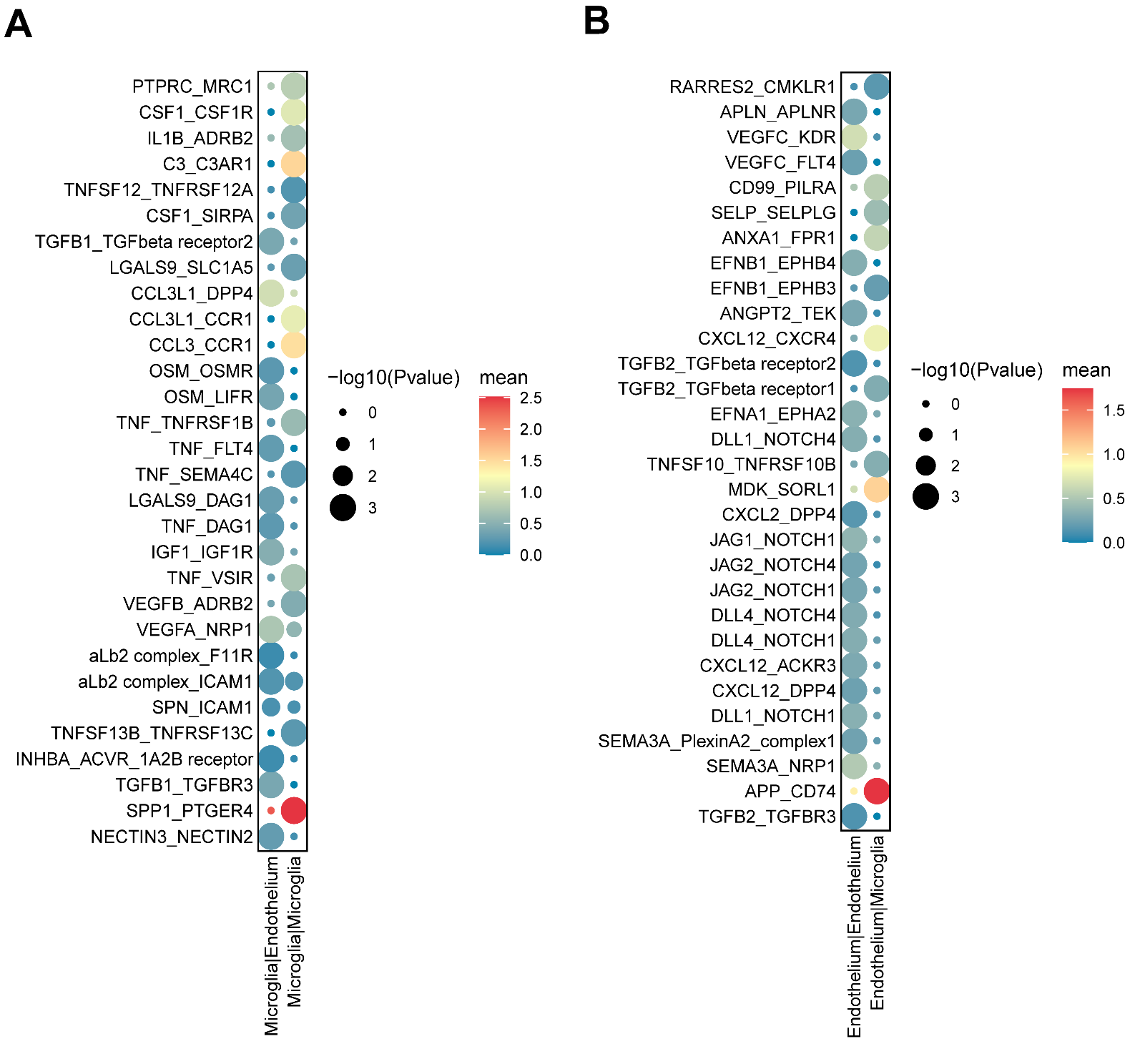
**

**Figure S2**


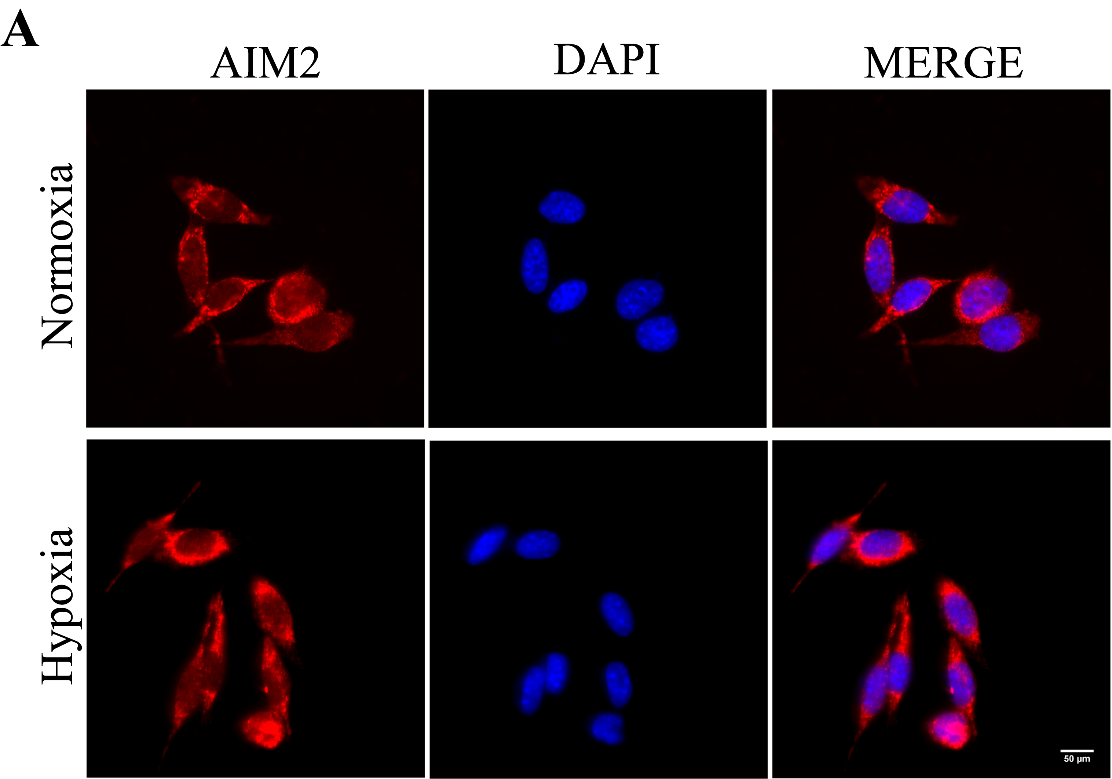


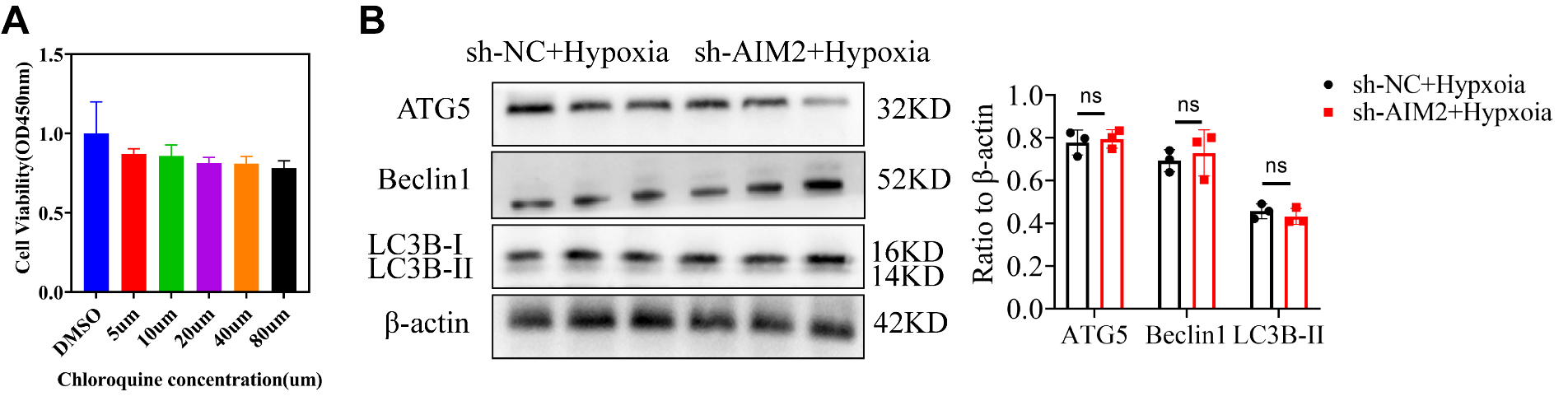
**Figure S3**


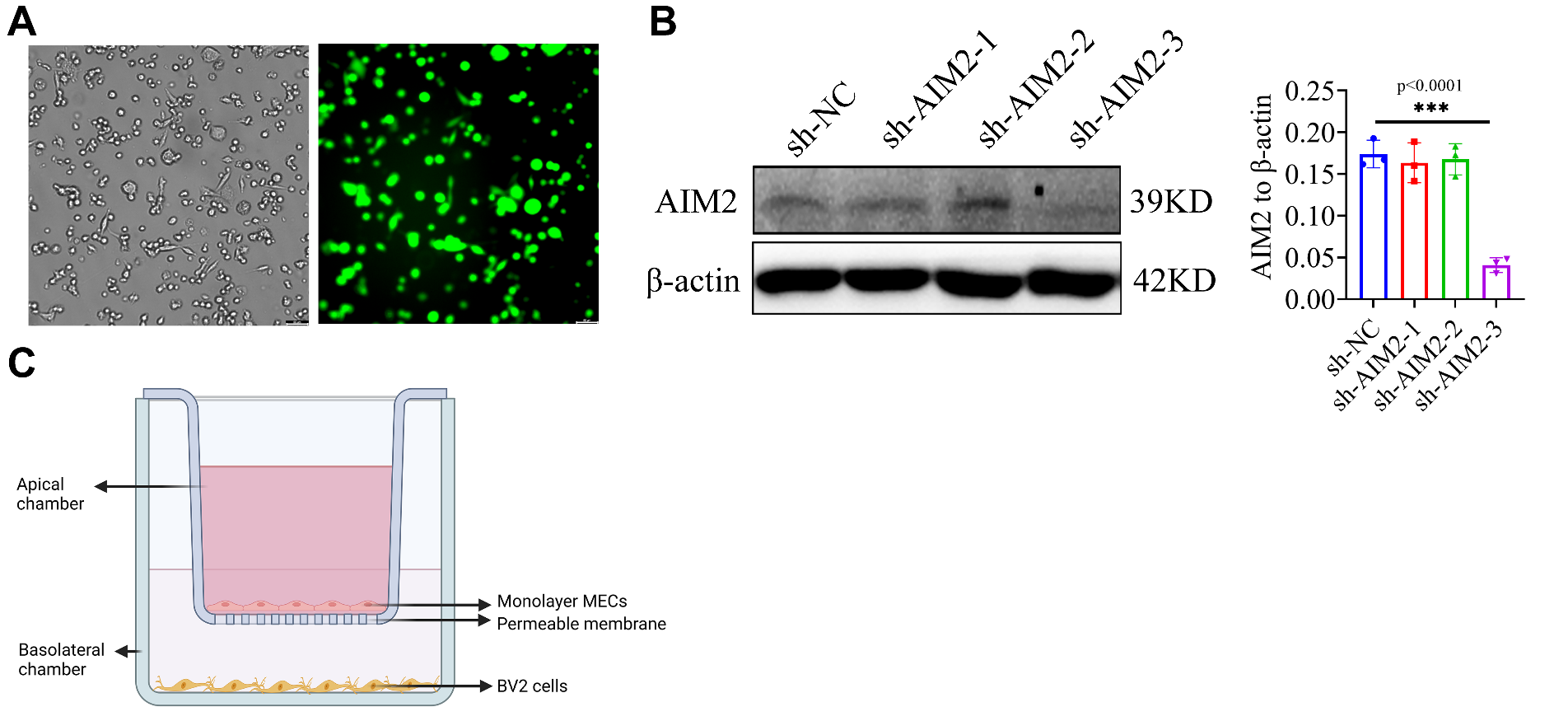
**Figure S4**

**Figure S5**


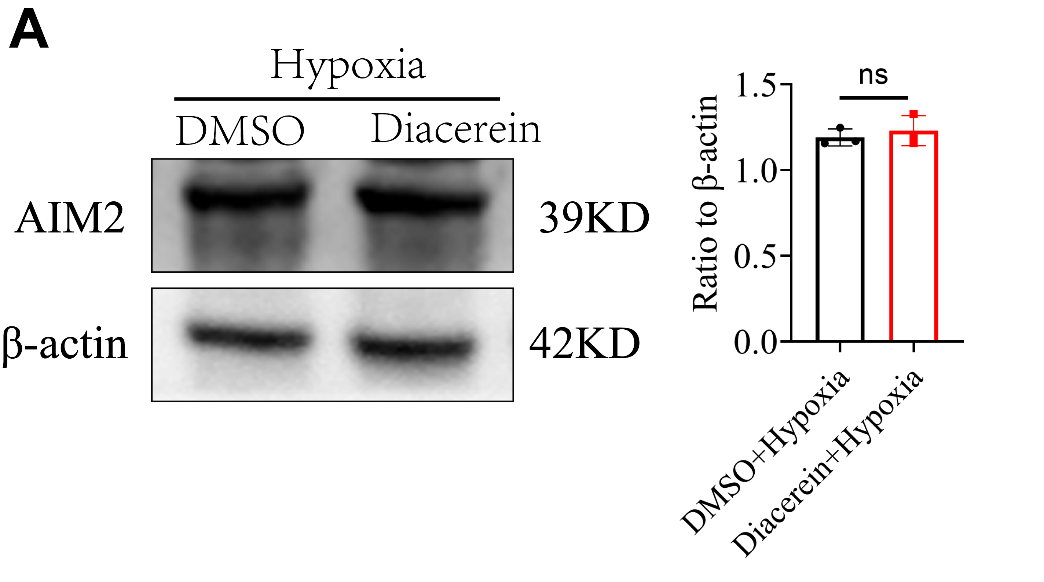


**Table S1. The Primers for RT-qPCR used in this study**

| **Genes** | **Species** | **Sequences (5’-3’)** |
| --- | --- | --- |
| *Nlrp1* | Mouse | GGTGGTGTGAAGATGTTGTGT |
|  |  | TCCATGTTCATCGTAGGGACC |
| *Nlrp3* | Mouse | ATTACCCGCCCGAGAAAGG |
|  |  | CATGAGTGTGGCTAGATCCAAG |
| *Nlrc4* | Mouse | ATCGTCATCACCGTGTGGAG |
|  |  | GCCAGACTCGCCTTCAATCA |
| *Nlrp6* | Mouse | CTCGCTTGCTAGTGACTACAC |
|  |  | AGTGCAAACAGCGTCTCGTT |
| *Nlrp12* | Mouse | GGATGGCCTCTATCGACTGTC |
|  |  | CCTCTGCAATCCCCAGGAATAA |
| *Aim2* | Mouse | GTCCTCAAGCTAAGCCTCAGA |
|  |  | CACCGTGACAACAAGTGGAT |
| *β-actin* | Mouse | GTGACGTTGACATCCGTAAAGA |
|  |  | GCCGGACTCATCGTACTCC |
|  |  |  |
